# Significant Liver-Related Morbidity After Bariatric Surgery and Its Reversal—a Case Series

**DOI:** 10.1007/s11695-017-2925-x

**Published:** 2017-09-30

**Authors:** Magdalena Eilenberg, Felix B. Langer, Andrea Beer, Michael Trauner, Gerhard Prager, Katharina Staufer

**Affiliations:** 1 Department of Surgery, Division of General Surgery, Medical University of Vienna, Vienna, Austria; 2 Department of Pathology, Medical University of Vienna, Vienna, Austria; 3 Department of Internal Medicine III, Division of Gastroenterology and Hepatology, Medical University of Vienna, Vienna, Austria; 4 Department of Surgery, Division of Transplantation, Medical University of Vienna, Waehringer Guertel 18-20, 1090 Vienna, Austria

**Keywords:** NAFLD, Weight loss, Liver dysfunction, Bypass reversal

## Abstract

**Background:**

Nonalcoholic fatty liver disease (NAFLD) occurs in up to 80% of patients with obesity. Current data suggest an improvement of NAFLD after established bariatric procedures.

**Objectives:**

This study investigated liver function impairment after Roux-en-Y gastric bypass (RYGB) and one-anastomosis gastric bypass (OAGB).

**Setting:**

University Hospital, Bariatric Surgery Unit

**Methods:**

In this single-center case series, consecutive in- and outpatients after bariatric surgery who presented with severe liver dysfunction from March 2014 to February 2017 were included and followed until March 2017.

**Results:**

In total, 10 patients (m:f = 2:8; median age 48 years, range 22–66 years) were included. Liver dysfunction occurred after a median postoperative time of 15 months (range 2–88 months). Median %excess weight loss at that time was 110.6% (range 85.2–155.5%). Liver steatosis/fibrosis occurred in 70%, cirrhosis in 30% of patients, and led to fatigue (90%), ascites (70%), hepatic encephalopathy (30%), and upper gastrointestinal bleeding (20%). Elevation of transaminases, impairment of coagulation parameters, thrombocytopenia, and hypoalbuminemia were present in 70, 80, 70, and 100%, respectively. In eight patients, lengthening of the alimentary/common limb led to an improvement or complete remission of symptoms. In one patient, liver transplantation was required, one patient deceased due to septic shock and decompensated liver disease.

**Conclusions:**

Severe liver dysfunction may also occur after bariatric procedures such as OAGB and RYGB. A comprehensive, meticulous follow-up for early identification of postoperative liver impairment should be aspired. Bypass length reduction led to a fast improvement in all patients.

## Introduction

Obesity and the concomitant metabolic syndrome represent a global health care issue. In up to 80%, obesity is associated with nonalcoholic fatty liver disease (NAFLD) including nonalcoholic steatohepatitis (NASH), which may progress to significant liver fibrosis, cirrhosis, and hepatocellular carcinoma [1]. Although lifestyle modification and weight loss is known to be the most effective treatment of NAFLD, long-term reduction of excessive overweight needs discipline and endurance, and is often unsuccessful.

During the last years, bariatric surgery has become an established procedure for effective and sustainable weight loss that may reverse or prevent long-term sequelae associated with obesity, such as cardiovascular disease, diabetes, musculoskeletal damage, and sleep apnea [2]. Importantly, in the majority of patients, bariatric surgery improves liver steatosis, inflammation, and fibrosis in NAFLD patients with obesity [3]. In up to 16% of cases, yet, an increase of liver fibrosis or de novo fibrosis is seen, but is consistently mild without development of cirrhosis or liver function alterations [4–10]. However, occasional deterioration of liver function was reported and may be attributed to the type of bariatric procedure and the extent of malnutrition and malabsorption. Jejunoileal bypass (JIB), and to a lesser extent also biliopancreatic diversion (BPD), are associated with a higher morbidity and mortality, particularly concerning liver function [11, 12]. Acute liver failure was reported in 7% in a study following 453 patients after JIB [13]. Therefore, nowadays, bariatric surgeons refrain from performing JIB.

To date, Roux-en-Y gastric bypass (RYGB) and sleeve gastrectomy (SG) are the most commonly performed procedures. The one-anastomosis gastric bypass (OAGB) or omega loop/mini gastric bypass is an up-and-coming procedure and is regarded as a technically simpler bypass accompanied by an increased %excess weight loss (%EWL), but very little complications [14, 15]. Clinical liver deterioration or histologic liver alterations after OAGB, SG, or gastric banding have not been reported so far.

In the present study, we aimed at highlighting the occurrence and clinical characteristics of liver dysfunction after RYGB, OAGB, and distal GB (performed due to failed weight loss or weight regain) as seen at our institution in a case series of 10 patients.

## Patients and Methods

Consecutive in- and outpatients after bariatric surgery who attended our facility at first presentation of liver dysfunction from March 2014 to February 2017 were included in the study and followed until April 2017. Patients’ health records were retrospectively screened for preoperative signs of liver disease and other comorbidities. All available pre- and postoperative results of liver imaging and liver histology, as well as the course of laboratory parameters and clinical symptoms as direct or indirect signs of liver dysfunction were analyzed. NAFLD was graded according to the NAFLD Activity Score (NAS). Additionally, clinical parameters such as age, sex, weight, body mass index (BMI), ∆BMI, %EWL, and % total weight loss (%TWL) [16] were documented prior to and/or after bariatric surgery, respectively. Obesity was graded according to the World Health Organization (WHO) classification [17]. The study was performed in accordance with the Declaration of Helsinki including current revisions.

## Results

### Demographics and Initial Body Weight

Patient characteristics are displayed in Table 1. Nine of 10 patients suffered from morbid obesity; one patient was classified as having obesity grade II according to the WHO classification [17]. No regular alcohol consumption or other liver diseases than NAFLD were present in any of the patients during the observed time period.

**Table 1:** Comparison of laboratory parameters prior to bariatric surgery and thereafter at the onset time of liver function deterioration

Study population, *n* = 10	Before bariatric surgery	Peak of liver dysfunction
Female sex, % (*n*)	80 (8/10)	80 (8/10)
Age in years, median (range)	40 (21–66)	48 (22–66)
BMI (kg/m^2^), median (range)	49.2 (38–64)	22.2 (20.8–30.5)
NAFLD		
Liver disease, % (*n*)	60 (6/10)	100 (10/10)
Steatosis/fibrosis, % (*n*)	50 (5/10)	70 (7/10)
Cirrhosis, % (*n*)	10 (1/10)	30 (3/10)
No liver disease	30 (3/10)	0 (0/10)
Missing, % (*n*)	10 (1/10)	0 (0/10)
Liver enzymes		
AST (U/L), median (range)	25 (14.5–57)	32.5 (14–258)
ALT (U/L), median (range)	38 (12.5–127)	26 (11–230)
GGT (U/L), median (range)	33 (12–110)	38.5 (11–468)
Hypercholesterolemia, % (*n*)	20 (2)	0 (0)
Cholesterol (mg/dl), median (range)	175.3 (109.5–228)	98.5 (66–146)
HDL (mg/dl), median (range)	52.5 (42–59)	42 (4–54)
LDL (mg/dl), median (range)	111.2 (92.1–152.8)	37.2 (11.2–92.2)
Hypertriglyceridemia, % (*n*)	20 (2)	10 (1)
Triglycerides (mg/dl), median (range)	92 (46–152)	50.5 (28–249)
T2DM, % (*n*)	10 (1)	0 (0)
OAD, % (*n*)	10 (1)	0 (0)
Insulin, % (*n*)	0 (0)	0 (0)
HbA1c (%), median (range)	5.2 (4.7–5.7)	3.9 (3.7–4)
aHTN, % (*n*)	40% (4)	10 (1)
Hyperuricemia, % (*n*)	20% (2)	0 (0)
Obstructive sleep apnea, % (*n*)	0 (0)	0 (0)
CAD, % (*n*)	0 (0)	0 (0)
Congestive heart failure, % (*n*)	10 (1)	10 (1)
PH, % (*n*)	10 (1)	10 (1)

*BMI* body mass index, *NAFLD* nonalcoholic fatty liver disease, *AST* alanin-aminotransferase, *GGT* gamma-glutamyl-transferase, *HDL* high-density lipoproteins, *LDL* low-density lipoprotein, *T2DM* type 2 diabetes mellitus, *OAD* oral antidiabetics, *aHTN* arterial hyptertension, *CAD* coronary artery disease, *PH* pulmonary hypertension

### Surgical Procedures and Weight Loss

In six of 10 patients (#1–5 and #8) surgery was performed at our center, whereas patients #6, 7, 9, and 10 were primarily operated elsewhere and transferred to our facilities only after complications had occurred. RYGB was the most frequently applied primary surgical procedure (*n* = 5). In three patients, OAGB and in two further patients gastric banding was performed (Table 2).

**Table 2:** Surgical procedures, clinical course, and treatment of all patients

Patient	Type of surgical intervention	Initial BMI (kg/m^2^)	BMI min (kg/m^2^)	ΔBMI (kg/m^2^)	%TWL (%)	%EWL (%)	Clinical presentation	Onset of symptoms (months)	Treatment
**1**	RYGB (BPL 60 cm, AL 200 cm, CL 200 cm)	48.2	30.00	18.2	37.7	78.3	No discomfort	84	
	Weight loss of unknown origin	48.2	21.90	26.3	54.6	113.3	Dysphagia, epigastric pain, diarrhea, sarcopenia, hypoalbuminemia, leg edema, thrombocytopenia, impaired coagulation, hepatomegaly	4	Gastric remnant feeding tube, GB reversal
**2**	Gastric band	38	18.40	19.6	51.6	155.5	Weight regain, dysphagia, band infection, pancytopenia, elevated liver enzymes	144	Band removal
	OAGB (BPL 175 cm, CL 500 cm)	33.3	22.03	11.3	33.8	125.4	Sarcopenia, ascites, HE, splenomegaly, variceal bleeding, leg edema, thrombocytopenia, impaired coagulation, hypoalbuminemia	5	LT
**3**	OAGB (BPL 370 cm, CL 320 cm)	42.9	20.80	22.1	51.5	123.7	Ascites, elevated liver enzymes, hepatosplenomegaly, pancytopenia	12	Conversion to RYGB, CL lengthening (BPL 220, AL 50 cm, CL420cm)
**4**	RYGB (BPL 60 cm, AL 150 cm CL200)	50.2	31.90	18.3	36.5	75.4	Weight regain	85	Distal GB
	distal GB (BPL 60 cm, AL 275 cm, CL 75 cm)	44.5	28.70	15.8	35.5	85.2	General fatigue, impaired coagulation, elevated liver enzymes, hypoalbuminemia, leg edema	12	Gastric remnant feeding tube, CL lengthening (BPL60cm, AL 150 cm, CL 200 cm)
**5**	RYGB (BPL 60 cm AL 200 cm, CL 425 cm)	44.1	22.70	21.4	48.5	111.8	Weight regain	96	Distal GB
	distal GB (BPL 400 cm, AL 200 cm, CL 85 cm)	28.1	20.30	7.8	27.8	124.6	Diarrhea, dumping syndrome, hypoalbuminemia, leg edema, impaired coagulation, SIBO, lactose malabsorption, hepatomegaly	6	CL lengthening (BPL 250 cm, AL 200 cm, CL 235 cm)
**6**	RYGB (limb length unknown)	58	28.70	29.3	50.5	88.8	Epigastric pain, elevated liver enzymes, thrombocytopenia, impaired coagulation, hypoalbuminemia, leg edema, ascites, HE, hepatosplenomegaly	2	Conservative
**7**	RYGB (BPL 155 cm, AL 210 cm, CL 35 cm)	53.4	26.60	26.8	50.2	94.4	Fatigue, ascites, leg edema, sarcopenia, hepatosplenomegaly, pleural effusions, hypoalbuminemia, elevated liver enzymes, thrombocytopenia	24	Gastric remnant feeding tube, CL lengthening (BPL 15 cm, AL 210, CL 175 cm)
**8**	OAGB (BPL 265 cm, CL 395 cm)	40.8	21.70	19.1	46.7	120.7	Fatigue, ascites, leg edema, sarcopenia, hypoalbuminemia, steatorrhea, diarrhea, thrombocytopenia, impaired coagulation, splenomegaly	35	Conversion to RYGB, CL lengthening to 530 cm (BPL 60, AL 70)
**9**	Gastric band	58.4	56.30	2.1	3.7	6.5	No weight loss	108	Band removal
	OAGB (BPL 200 cm, CL 375 cm)	57.6	36.80	20.8	37.0	64.7	Diarrhea, SIBO	36	
	Weight loss of unknown origin	57.6	22.40	35.2	61.7	107.8	Dysphagia, epigastric pain, elevated liver enzymes, sarcopenia, pleural effusions, ascites, hypoalbuminemia, HE, steatorrhea, diarrhea	5	Conversion to RYGB, CL lengthening to 495 cm (BPL 30 cm, AL 50 cm)
**10**	OAGB (BPL 95 cm, CL 275 cm)	64	30.5	33.5	52.4	85.9	Fatigue, dysphagia, ascites, leg edema, pulmonary effusion, sarcopenia, hypoalbuminemia, jaundice, thrombocytopenia, elevated liver enzymes, impaired coagulation, hepatosplenomegaly	20	Conversion to RYGB, CL lengthening to 295 cm (BPL 35 cm, AL 40 cm)

*BMI*, body mass index, *ΔBMI* (initial BMI) − (minimal BMI), *%TWL* percent of total weight loss ([(initial weight) − (postop weight)]/[(initial weight)] × 100), *%EWL* percent excess weight loss ([(initial weight) − (postop weight)]/[(initial weight) − (ideal weight)], *GB* gastric bypass, *BPL* biliopancreatic limb, *AL* alimentary limb, *CL* common limb, *RYGB* Roux-en-Y GB, *OABG* one-anastomosis GB, *BMI* body mass index, *%EWL* percentage of excess weight loss, *HE* hepatic encephalopathy, *SIBO* small intestinal bacterial overgrowth, *LT* liver transplantation

In one patient (#9) no successful weight loss was achieved after gastric banding (6.5%EWL), so that after band removal an OAGB was performed. In the further nine patients, the primary surgical procedure led to a significant weight reduction of 111.8% median %EWL (range 75.4–155.5%; median BMI 22.7 kg/m^2^, range 18.4–31.9 kg/m^2^; for ΔBMI and %TWL (%), see Table 2). However, in three of these patients (#2, 4, and 5), a weight regain occurred after a median time of 7 years (range 1–8 years), in one patient after removal of gastric band due to dysphagia and in two patients after RYGB. Therefore, a conversion to OAGB in the first patient and a shortening of the common channel for reduction of the absorption length in the two other patients were performed (after exclusion of preexisting food restriction). In total, four of 10 patients required a secondary procedure for persistent weight reduction.

Including all primary and secondary surgical procedures, the overall patient’s lowest median BMI was 22.2 kg/m^2^ (range 18.4–30.5 kg/m^2^) which was equivalent to a median %EWL of 110.6% (85.2–155.5%, and median %TWL of 51.6%, range 42.0–61.7%). The median remaining intestinal resorption length (alimentary plus common limb) was 357.5 cm (range 245–500 cm).

### Liver Alterations and Course of Liver Deterioration

**Fig. 1:**
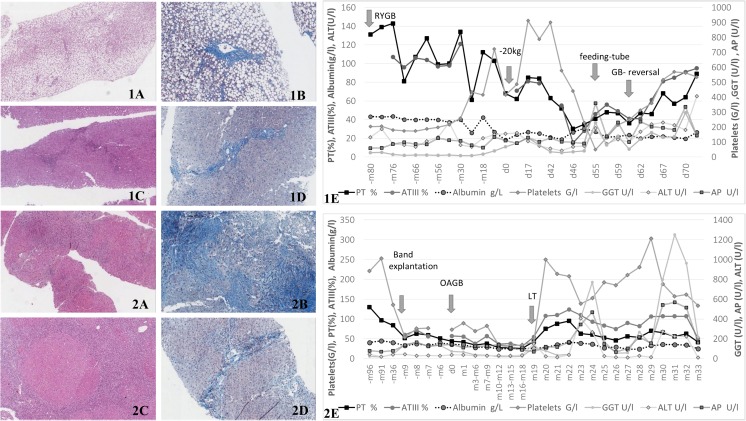
Histopathological findings of liver biopsy and the corresponding clinical course of patient 1 and 2. Patient #1 (1A–E): 1A (hematoxylin-eosin [HE] staining, ×8 magnification [mag.]) and 1B (chromotrop-aniline blue [CAB] staining, ×20 mag.): histology gained at time of feeding-tube implantation; steatosis (95% macrovesicular, 5% microvesicular) with partly periportal fibrosis und minor inflammatory activity, correlating with NASH, NA-Score: 3-1-1 (5/8), fibrosis grade 1C. 1C (HE staining, ×10 mag.), and 1D (CAB staining ×20 mag.): histology gained 5 months after bypass reversal: portal and periportal fibrosis, incipient portoportal septation. No micro- or macrovesicular steatosis, no inflammatory activity, no hepatocellular ballooning. 1E: laboratory-values and clinical events over time. Patient #2 (2A–E): 2A (HE staining, ×8 mag.): histology gained after explantation of the gastric band: liver tissue with broad, septal fibrosis and starting, focal cirrhotic alteration, no inflammatory activity, corresponding to resolved NASH. 2B (CAB staining, ×20 mag.): histology gained after OAGB: focal cirrhosis, pericellular fibrosis, and moderate steatosis (20% micro-, 5% macrovesicular). 2C (HE staining, ×8 mag.): histology gained at time of liver transplantation (LT): liver cirrhosis and siderosis, marginal irregular steatosis (10% microvesicular), Ludwig-Score: portal: 2, lobular: 1, fibrosis grade 4. 2D (CAB staining, ×20 mag.): histology gained 4 months after LT: hepatic picture with minor inflammatory activity, cholestasis, and focal portoportal fibrosis. 2E: laboratory values and clinical events over time

The features of liver alterations and clinical presentation are displayed in Tables 1 and 2, as well as Fig. 1. Liver dysfunction developed after a median interval of 15 months (range 2–88 months), after performing OAGB in four patients (#2, 3, 8, and 10), after RYGB in two patients (#6 and 7), and after distal GB following RYGB in two patients (#4 and 5). In the remaining two patients, liver dysfunction occurred after a symptom-free period of weight stability (84 months after RYGB in patient #1 and 36 months after OAGB in patient #9) followed by a further significant weight loss of unknown origin. Four and 5 months thereafter, signs of malnutrition and liver dysfunction became evident (Table 2).

Liver dysfunction was mainly characterized by a moderate increase in liver enzymes (70%), hepatosplenomegaly (80%), thrombocytopenia (70%), impaired coagulation parameters (80%), hypoalbuminemia (100%), and sarcopenia (60%). At later stages, patients suffered from ascites (70%), pleural effusions (30%), hepatic encephalopathy (30%), hepatorenal syndrome (10%), and upper gastrointestinal bleeding (20%).

The referring histologic picture of liver alterations after bariatric surgery was available in seven patients. While there was a 90 to 100% micro- and macrovesicular steatosis in two patients (#1 and 9), in three others a cirrhotic remodeling was predominant (#2, 3, and 8). In patient #7 and #10, a worsening from no comprehensible pathology to macrovesicular steatosis (#7:20%; #10:85%) and fibrosis (#7:grade 1a, NAS 3/8; #10:grade 3; NAS 7/8) was documented. In the other three patients’ (#4, 5, and 6) sonography and/or computed tomography showed steatosis and hepatosplenomegaly, as well as ascites (#6).

As a consequence, after bridging by implanting a feeding tube into the remnant stomach to increase absorption (#1, 4 and 7), the common channel was lengthened (at the expense of the biliopancreatic limb) in seven patients (#3–5, and #7–10) and the entire bypass reversed in one patient (#1). In patient #2, ultimately, liver transplantation was required. Patient #6 was treated conservatively and died due to septic shock and decompensated liver disease prior to any surgical conversion could have been performed.

Of note, after the original intestinal anatomy was restored in patient #1, a clinical stabilization occurred as assessed by laboratory parameters (Fig. 1, 1A–E). Furthermore, a complete reversal of a prior 100% steatosis was found and documented by liver biopsy (fig. 1, 1D). In patients #3 to 5 and #7 to 10, the lengthening of the common channel at expense of the biliopancreatic limb led to a clinical stabilization and significant improvement both in imaging and laboratory parameters in all patients (data not shown). In patient #2 (Fig. 1, 2A–E) who received a liver transplant only 11 months after liver transplantation again, transaminases increased to six times the upper limit of normal. Performing a re-conversion operation was considered too risky in view of a common limb of 500 cm and a questionable benefit of the reversal since liver biopsy additionally revealed de novo post-transplantation autoimmune hepatitis (Fig. 1, 2D).

## Discussion

In the present study, we report 10 cases of significant liver dysfunction after OAGB, RYGB, and distal GB for weight loss failure or weight regain following RYGB. Liver dysfunction occurred after a median time period of 15 months after surgery had been performed. In two of 10 patients, liver dysfunction developed after a significant additional unexplained weight loss occurring after a long time period of stable weight after RYGB or OAGB. Total median %EWL was 110.6% in all patients. In two thirds of patients, NAFLD was present already prior to surgery, but aggravated thereafter. In two patients, liver disease developed only after surgery.

In general, bariatric surgery has been successfully utilized to reverse or prevent further progression of NAFLD [18–22]. In a large meta-analysis, comprising 766 patients receiving paired liver biopsies after BPD, gastric banding, or RYGB, improvement or resolution of steatosis, steatohepatitis, and fibrosis was reported in 91.6, 81.3, and 65.5% of patients, respectively [3]. In contrast, although an improvement in steatosis and hepatic cell ballooning may be observed within the first year after these procedures, in a prospective study including 381 patients, although 95.7% maintained a fibrosis score ≤ F1, fibrosis had progressed significantly after 5 years, and was associated with higher BMIs and increased insulin resistance [9]. However, apart from only slight impairment of liver histology, rarely, also severe deterioration leading to liver failure and death was described predominantly after JIB or BPD [12]. In contrast, there are only few single cases of significant liver dysfunction after laparoscopic RYGB [23]. To the best of our knowledge, there have been no reports of OAGB leading to a moderate or even excessive deterioration of liver function to the requirement of LT, as it was seen in our study.

The underlying mechanisms for liver deterioration after bariatric surgery have not yet been understood and prognostic surrogate parameters are lacking. It has been shown that purely restrictive procedures such as SG are feasible and effective also in patients with preexisting advanced liver disease [24]. To the best of our knowledge, severe liver deterioration after SG or gastric banding has not been described so far. In contrast, very recently, a randomized trial reported that patients with NASH undergoing RYGB were more susceptible to early transient deterioration of liver function compared to SG [25].

A negative impact on liver function after RYGB has been suggested to occur due to extended excluded limbs or distal versions of RYGB [26]. In our series, the dimensions of the common channels (median length 357.5 cm) should have been more than sufficient for adequate absorption. Nevertheless, although BMI remained within a normal range, median %EWL was significantly higher than average. It seems therefore apparent that an adequate limb length does not guarantee appropriate absorption in the individual patient. In fact, differing adaptation of the intestinal mucosa has been hypothesized [27].

Negative events after RYGB were furthermore reported to be largely associated with preoperatively diagnosed cirrhosis, alcohol abuse, and intraoperative complications [28]. The degree of liver disease, yet, had no effect on the perioperative outcome, but indicated an association with higher mortality in patients suffering from NASH at long-term follow-up [29, 30]. A systematic review in 108 mainly Child Pugh A patients undergoing bariatric surgery suggested an increased morbidity and mortality in these patients and described a decompensation of liver disease in six cases, as well as death due to fulminant hepatic failure in four cases [28]. This may lead to the question whether patients with a preexisting liver disease are more susceptible to a “second hit” due to rapid weight loss or undefined factors caused by malabsorptive procedures.

In our case series, both the facts that 60% of our patients showed preexisting liver disease and that a high total median %EWL of 110.6.3% occurred may have contributed to patients’ liver dysfunction. Importantly, the OAGB which is considered a safe procedure has led to a considerate liver impairment in five of our patients [31]. By elongation of the common limb or BP reversal, a significant improvement or even cessation of symptoms could be achieved in all patients after RYGB or OAGB.

Of note, up to 5% of patients with morbid obesity patients already suffer from undiagnosed cirrhosis at the time of bariatric surgery [2]. Symptoms can be relatively unspecific and even intraoperatively macroscopic liver disease may be easily overlooked [32]. In consequence, the presence of advanced liver disease should be thoroughly excluded prior to bariatric surgery. The importance of a meticulous postoperative follow-up including close monitoring of liver function tests needs to be emphasized. The focus might especially be brought on patients with previous morbid/super obesity, continuously impaired insulin resistance, and preoperative liver pathologies, as well as sudden and fast weight loss occurring independently from the primary procedure until prospective factors for postoperative liver deterioration have been established.

Large prospective clinical and basic research studies are required for a better understanding of the extent of our observation in this patient collective and for the determination of possible origins behind a deterioration of liver function after bariatric surgery.

### Limitations

Due to the retrospective nature of this study, we cannot give information on the overall prevalence of liver deterioration after bariatric surgery. Diagnostic methods were adapted to the clinical appearance of symptoms in each individual patient, and the prevalence of liver dysfunction might be underestimated.

## Conclusion

To date, there are no recommendations for handling bariatric patients suffering from a certain degree of liver disease as there is insufficient evidence, so far. Considering the fact that obesity comes in hand with a high and increasing prevalence of NAFLD, guidelines for pre- and postoperative surveillance are warranted. We need to stress that in our cases, a very mild preoperative liver pathology has led to a clinically critical liver disease after the less malabsorptive procedures of OAGB and RYGB. In the presented cases, elongation of the resorption length of the bypass has led to an improvement in symptoms in all patients and this might be considered in patients showing a similar pattern of liver dysfunction after a bariatric procedure.
